# Single-Exosome SERS Detection by Means of a Flexible Metasurface

**DOI:** 10.3390/bios15120815

**Published:** 2025-12-15

**Authors:** Konstantin Mochalov, Denis Korzhov, Milena Shestopalova, Andrey Ivanov, Konstantin Afanasev, Alexander Smyk, Alexander Shurygin, Andrey K. Sarychev

**Affiliations:** 1 Shemyakin–Ovchinnikov Institute of Bioorganic Chemistry, Russian Academy of Sciences, 16/10 Miklukho-Maklaya Street, Moscow 117997, Russia; mochalov@mail.ru (K.M.); shestopalova.milena@icloud.com (M.S.); 2 Educational Resource Center for Cellular Technology, RUDN University, 6 Miklukho-Maklaya Street, Moscow 117198, Russia; 3 Department of Micro and Nano Systems, National Research Nuclear University MEPHI, Kashirskoe Shosse 31, Moscow 115409, Russia; 4 Department of Solid-State Physics and Nanosystems, National Research Nuclear University MEPHI, Kashirskoe Shosse 31, Moscow 115409, Russia; 5 Institute for Theoretical and Applied Electromagnetics, Russian Academy of Sciences, 13/6 Izhorskaya Street, Moscow 125412, Russia; av.ivanov@physics.msu.ru (A.I.); kavacuum@mail.ru (K.A.); 6 James River Branch LLC, Tvardovsky St., 8, Moscow 123458, Russia

**Keywords:** biosensor, SERS, extracellular vesicles, exosomes, metasurface, plasmon

## Abstract

Single exosomes are detected via surface-enhanced Raman scattering (SERS) due to electromagnetic field accumulation on a specially designed flexible metasurface. This metasurface is a modulated silver nanofilm deposited on a thin, flexible plastic substrate. An explicit Equation for calculating the local electric field is given. The field reaches extremely high values under plasmon resonance conditions and fills the depressions of the metasurface. The thin, flexible metasurface can be incorporated into automated Lab-On-Chip analytical systems and used for spectroscopic studies of exosomes. We propose a method to distinguish individual exosomes from the HEK293T cell line on the metasurface and then obtain and assign their SERS spectra. An important advantage of the plasmonic metasurface presented in this work is its spatial complementarity to exosomes and other vesicle-like objects. The plasmonic metasurface is fabricated using holographic lithography and further investigated using a correlation approach combining atomic force microscopy, scanning spreading resistance microscopy, and surface-enhanced spectroscopy.

## 1. Introduction

Single exosomes are detected via a Raman signal from a metasurface (MS) specially developed for research. Exosomes are extracellular vesicles with a diameter of a∼ 100 nm. They can reflect the biological information of their parent cells and participate in intercellular communication [1]. Exosomes have become one of the most promising markers for the early diagnosis and prognosis of cancers and other diseases. With the development of modern optical technology and the emergence of high-resolution optical microscopies, the micro/nanostructures of exosomes can be observed optically for the first time. A flexible metasurface (FMS) is being developed to obtain Raman signals from single exosomes. A plasmon FMS accumulates energy from incident light by exciting local plasmon resonators. Energy storage in plasmon resonances generates a giant local electric field, which is important for photovoltaics, photodetectors [2,3], and metalenses [4,5]. MSs are used for near-field power transfer [6]; generation of second, third, and higher harmonics from an incident laser beam [7,8]; and surface-enhanced Raman scattering (SERS) [9,10]. Recently developed MSs generate hotspots with a local photonic density of states, which, in principle, allows the detection of microscopic quantities of biomolecules and bioobjects. Metal–dielectric MSs, which use gold or silver as metal components [11,12,13,14], exhibit stability and reproducibility. Gold or silver FMSs can be integrated into a microfluidic analytical system, thus creating a so-called Lab-On-Chip for medical diagnostics [15,16,17,18,19,20,21,22]. Clinical microfluidic platform for rapid label-free capture and SERS detection of viruses has been reported [23]. A nanoimprinted lithographic FMS was developed for SERS sensors for SARS-CoV-2 viruses [24]. Recently, [25] an MS composed of hydrophobic silver nanowires (H-CuO@Ag NWs) was used for the quantitative detection of trace nanoplastics (down to a∼ 50 nm) via SERS based on a multiplex-feature coffee ring approach. The experimental results revealed a strong correlation between the formation of the coffee ring and the concentration of the nanoplastic dispersion. The authors developed a machine learning model that maps the nanoplastic concentration to the coffee ring characteristics, and plastic nanoparticles in natural river water were detected. The close similarity in SERS detection of nanoplastics and exosomes should be noted. EM field enhancement in an open resonator (e.g., [26]) can be also invoked.

Exosomes are nanosized lipid droplets, which, as suggested in the modern literature [27,28], are formed by living cells for intercellular communication. Exosomes include protein, RNA, and DNA molecules, so they are assumed to participate in the regulation of normal as well as pathological processes in living cells [29,30,31]. Exosomes are intensely studied since they are promising agents for oncology diagnostics [32,33]. Raman and IR spectroscopy are employed for the study of exosomes (see, e.g., [1]). Raman scattering (RS) has a very small scattering cross-section, and to obtain a pronounced RS, it is necessary to collect a large number of exosomes and obtain a collective RS signal, including traces of exosome compaction. A solution is the study of the exosomes using the SERS method, which enhances RS by many orders of magnitude [34,35]. SERS technology is highly relevant to exosome detection due to its unique ability to provide highly sensitive and specific molecular fingerprints. SERS can be employed to detect exosomes with minimal sample preparation, making it an ideal method for analyzing exosomes, including detection of various proteins, nucleic acids, and lipids [34,35,36,37,38,39,40].

Numerous researchers have employed SERS for the identification of exosomes, utilizing methodologies that can be classified into labeled and label-free approaches. Labeled techniques offer high accuracy and semi-quantitative analysis; however, they are limited to capturing information from the labels [41,42,43,44,45,46]. For example, nanoprobes and magnetic nanobeads were used in [41] for SERS-based detection of tumor-derived exosomes. Magnetic nanobeads and SERS nanoprobes can capture exosomes by forming a sandwich-type complex. The exosome–nanobead complexes are precipitated with a magnet, and thus SERS signals are detected from compacted exosomes.

Label-free SERS detection of exosomes requires simultaneous detection of different exosomes. Label-free SERS, in principle, allows one to distinguish between various exosomes. In the seminal work [47], small drops of solution containing exosomes, obtained from either healthy or tumor colon cells, were deposited on a super-hydrophobic surface consisting of periodic silicone micropillars, the tops of which are deposited with silver nanoparticles (NPs). SERS spectra obtained from healthy or tumor colon exosomes are found to be somewhat different. A direct Raman signal was obtained in [48] from purified exosomes. This signal allows one to distinguish exosomes derived from seven cancerous and non-cancerous cell lines. In [49], it is shown that regular recordable disks (CD-R and DVD-R) coated with a silver nanolayer reveal the SERS fingerprint of hemoglobin and exosomes. In [50], gold–silver NP shells were formed around exosomes to obtain their SERS spectra. In a series of investigations [51,52,53], SERS spectra of individual exosomes were obtained using a hybrid SERS substrate, which comprised a graphene-covered quasi-periodic array of gold pyramids (see also simulations in [54]). SERS spectra of lung cancer exosomes were also obtained from graphene-covered gold pyramids [52]. A hybrid porous biomaterial scaffold with silver NPs is suggested in [55] for a liquid biopsy via exosome diagnostics. Acoustofluidics-induced enrichment of NPs is introduced in [55]. CD63 aptamer-conjugated silica NPs + exosomes were pushed by acoustic waves to the surface of the glass capillary covered by ZnO nanorods, which was analyzed via SERS. In [56], $TiO_{2}$ micro-porous inverse opal honeycomb coated by gold nanofilm was used as an effective SERS substrate. The honeycomb structure captures the exosomes from the plasma of cancer patients. The authors focused on the intensity of the 1087 cm^−1^ Raman line, which is a signature of phosphoproteins. They speculated that the intensity of the line is a signature of tumor exosomes. In important works by Shin et al. [57,58] gold NPs with a size of a∼ 100 nm were settled onto glass, where they formed fractal clusters. The fractal substrate was used for exosome SERS characterization and detection of six types of early-stage cancers (lung, breast, colon, liver, pancreas, and stomach). A microfluidic device with SERS for the identification of glioblastoma exosomes is considered in [38]. The same group developed a multiplex fluidic device with embedded arrayed nanocavity microchips that achieves confinement of single exosomes in a minute amount of fluid (<10 μL) and provides the SERS spectrum [59]. In [60], a nano-engineered 3D SERS sensor was reported. The sensor is composed of titania ($TiO_{2}$) NPs (a∼ 10 nm) containing SERS-active Magneli black $TiO_{2}$. An ultralow limit of detection (10 exosomes per 10 μL) was achieved. The achievement is attributed to the simultaneous detection of multiple exosome signals. The paper [61] proposes rapid and label-free detection of breast-cancer-derived exosomes from SERS spectra obtained via the laser ablation of a silver plate. In [62], SERS was performed with the combination of Ag NPs and a single atomic layer of graphic carbon nitride $C_{3}N_{4}$, which concentrates exosomes due to the π−π interaction between aromatic exosome molecules and the carbon nitride. In [63], exosomes were investigated using tip-enhanced Raman spectroscopy. Contact between the tip and the bovine milk exosomes can be used for drug delivery and therapeutic applications. In [64], SERS from the nanowire microlens was used for cancer diagnosis, and 24 cases of early pancreatic cancer were successfully classified. In [65], it was proposed to perform exosome detection via SERS based on a TiN–Ag@Ag sol composite substrate. A statistical difference was obtained in the SERS of exosomes extracted from the serum of 31 gastric cancer patients and 31 healthy controls.

To our knowledge, there are currently no generally accepted solutions for exosome diagnostics. For example, microRNA from exosomes, which is supposed to serve as a tumor marker, varies in different studies (cf. studies of colorectal cancer [66,67]). The membrane proteins of exosomes may be candidates for the role of tumor markers. The tumor marker proteins HER2, EpCAM, CD63, and PSMA have been proposed for the diagnosis of breast and pancreatic cancer [68,69,70,71,72,73,74].

As we mentioned above, there are two approaches to SERS studies. Metal NPs can be added to an exosome solution in such a way that NPs and exosomes form a mixed aggregate [41,43,44,45,75,76]. Collective plasmon resonances in the metal fractal aggregate lead to an increase in the local electric field *E*. This field interacts with the exosomes incorporated in the aggregate. The exosomes, in turn, generate Raman signals that excite secondary plasmon resonances in the aggregate of metal NPs [77,78]. The emission of secondary plasmons enables SERS, the amplitude of which, *G*, is proportional to the fourth power of the local field $G∼E^{4}$ due to the two-stage scattering. The local field is significantly enhanced in the gaps between NPs. However, the gaps between NPs should be much smaller than the particle size (see, e.g., the explicit equation in [79] and the discussion therein). In any case, the NP+exosome aggregate facilitates collective SERS from captured exosomes.

We believe that plasmon MSs are promising tools for obtaining SERS signals from exosomes [79,80,81]. In the near future, the efforts of dozens of experimental groups around the world could allow us to diagnose cancer and other diseases from the SERS spectra of exosomes extracted from liquid biopsies. An important step in this direction is to obtain spectra of individual exosomes. In this paper, we present the Raman spectra of single exosomes deposited on a specially designed silver film, which is modulated with a period ≈ 400 nm smaller than the size of an exosome. An exosome at the smooth silver surface can be visualized using, for example, atomic force microscopy (AFM) as discussed below. An explicit equation is suggested for finding the local electric field in this FMS. The modulation is specifically designed to localize the plasmon in the FMS recesses, where the exosomes are typically localized. The structure of the plasmon electric field appears to be optimal for SERS studies of single exosomes. The fabrication process of the FMS, suitable for mass production, is discussed. The method was specially developed for exosome deposition. The resulting FMS with placed exosomes was used to obtain the Raman spectra of individual exosomes.

## 2. Materials and Methods

### 2.1. Theoretical Background

A thin, modulated, flexible metal film that operates as a flexible metasurface (FMS) is used to detect the Raman spectrum of an exosome or any other microscopic object. This FMS effectively concentrates the electromagnetic field and operates as an SERS substrate [22,80]. To understand the plasmon resonance and the field enhancement in the FMS, we consider the plasmon resonance in the simplest plasmon system, namely, the metal hollow nanocylinder (NC) with an internal radius $r=r_{i}$ and external radius $r=r_{e}$. The thickness $d=r_{e}-r_{i}$ of the NC is assumed to be less than the skin depth $\delta ∼{\left(kℑ\sqrt{\varepsilon_{m}}\right)}^{-1}\gg d$, where k=ω/c is a wavevector, ω is the frequency of the external electric field, and $\varepsilon_{m}\left(\omega \right)$ is the metal permittivity. It is also assumed that $r_{e}k\ll 1$, though it is not so critical for the application of the quasistatic approximation. The NC is excited by an external electric field. The electric field, calculated in the quasistatic approximation, has a textbook form [82] when the NC is excited by the uniform external field $E_{0}$. The field inside the hollow is uniform, and it is equal to $E_{i}=E_{0}A/Det,$ where the coefficient $A=4\alpha^{2}\varepsilon_{e}\varepsilon_{m}$, and the determinant is equal to

(1) $$Det=\alpha^{2}\left(\varepsilon_{e}+\varepsilon_{m}\right)\left(\varepsilon_{i}+\varepsilon_{m}\right)+\left(\varepsilon_{m}-\varepsilon_{e}\right)\left(\varepsilon_{i}-\varepsilon_{m}\right),$$

where $\alpha =r_{e}/r_{i}$; $\varepsilon_{i}$, $\varepsilon_{m}=\varepsilon_{m}^{\prime }+{i\varepsilon }_{m}^{\prime \prime };$ and $\varepsilon_{e}$ are the permittivities of the internal dielectric, metal NC, and the ambient medium, respectively. The optical metal permittivity $\varepsilon_{m}\left(\omega \right)$ depends on the frequency, and its real part $\varepsilon_{m}^{\prime }\left(\omega \right)<0$ is typically negative in optics. The imaginary part $\varepsilon_{m}^{\prime \prime }$ of the metal permittivity is small for good optical metals like silver or gold, and the optical quality $Q_{m}=\left|{\varepsilon^{\prime }}_{m}\left(\omega \right)\right|/\varepsilon_{m}^{\prime \prime }\left(\omega \right)$ is large [83]. For any given frequency ω, there is a critical ratio $\alpha =r_{e}/r_{i}$ of the external $r_{e}$ and the internal $r_{i}$ radii of the cylinder

(2) $$\alpha_{Res}≃\sqrt{\frac{\left(\varepsilon_{e}-\varepsilon_{m}^{\prime }\right)\left(\varepsilon_{i}-\varepsilon_{m}^{\prime }\right)}{\left(\varepsilon_{e}+\varepsilon_{m}^{\prime }\right)\left(\varepsilon_{i}+\varepsilon_{m}^{\prime }\right)}},$$

when Det is minimal in absolute value,

(3) $$Det_{Res}≃\frac{2i\left(\varepsilon_{e}+\varepsilon_{i}\right)\varepsilon_{m}^{\prime }\left({\varepsilon_{m}^{\prime }}^{2}-\varepsilon_{e}\varepsilon_{i}\right)}{Q_{m}\left(\varepsilon_{e}+\varepsilon_{m}^{\prime }\right)\left(\varepsilon_{i}+\varepsilon_{m}^{\prime }\right)}≃\frac{2i\left(\varepsilon_{e}+\varepsilon_{i}\right)\varepsilon_{m}^{\prime }}{Q_{m}},$$

where it is assumed that the optical quality $Q_{m}\left(\omega \right)\gg 1$; the last estimate in the above equation holds when $\left|\varepsilon_{m}^{\prime }\right|\gg 1$, which is typical for red and infrared spectral ranges. Then, the internal field $E_{i}$ is much enhanced: $\left|E_{i}/E_{0}\right|≃4\varepsilon_{e}\alpha_{Res}^{2}\varepsilon_{m}^{\prime }/Det_{Res}≃2\varepsilon_{e}Q_{m}/\left(\varepsilon_{e}+\varepsilon_{i}\right)∼Q_{m}\gg 1.$

When a molecule is placed inside the NC, it generates a Raman signal at the Stokes frequency $\omega_{s}\approx \omega$, which in turn excites the secondary plasmon resonance in the cylinder. The sequential plasmon resonances exhibit an effect called surface-enhanced Raman scattering (SERS). The total Raman enhancement is estimated as $G\;∼Q_{m}^{4}\gg 1$ since the Stokes frequency $\omega_{s}$ is typically close to the laser frequency $\omega_{s}\approx \omega$.

Let us consider the internal plasmon resonance excited from within the hollow NC. To calculate the local electric field, it is convenient to use the complex potential *U*, while its real part has physical meaning. We measure all distances in terms of the internal radius $r_{i}$, and we introduce dimensionless complex coordinates *u* and *v* with the origin in the center of the NC and complex variable w=u+iv. Then, in terms of the dimensionless coordinate *w*, the internal and external surfaces of the NC are given by $w_{i}=\exp\left(i\varphi \right)$ and $w_{e}=\alpha \exp\left(i\varphi \right)$, respectively, where $\alpha =r_{e}/r_{i}$ and φ are the polar angles in the *u*-*v* coordinate system.

Suppose the potential source, which is placed inside the NC, is equal to $U_{ext}=iU_{0}\log\left(w-a\right)$, where the parameter 0<a<1. The exact nature of this source is not essential for further conclusions. The potential of the source can be expanded in a series of power a/w

(4) $$U_{ext}=iU_{0}\log\left(w-a\right)=iU_{0}\log w-iU_{0}\sum_{n=1}\frac{1}{n}{\left(\frac{a}{w}\right)}^{n},$$

where we suppose that the radius $r=\left|w\right|$ is larger than the source coordinate r>a. The first term in Equation (4) gives the circular electric field, the amplitude of which, ∼1/r, does not depend on metal permittivity. The second, third, etc., terms in Equation (4) excite dipole, quadrupole, etc., plasmon resonances in the NC. The first term in the sum in the r.h.s. of Equation (4) is equal to $U_{d}=-iU_{0}a/w$, and it excites the electric field with the potentials $U_{i}=Aw$, $U_{m}=Bw+C/w$, and $U_{e}=D/w$ inside the hollow NC (r<1), in the metal NC (1<r<α), and in the external space (r>α), respectively. The radial $E_{r}$ and the polar $E_{\varphi }$ electric fields are obtained from the complex potential as follows: $E_{r}-iE_{\varphi }=-\exp\left(i\varphi \right)dU/dw$. The coefficients A, B, C, and D are found by matching the polar electric fields and normal electric displacement at the inner and outer interfaces of the NC. The amplitude of the internal “dipole” electric field is constant and is equal to

(5) $$\left|\frac{1}{U_{0}}\frac{d\;U_{i}}{d\;w}\right|=\left|A\right|=\frac{a\left|\alpha^{2}\left(\varepsilon_{e}+\varepsilon_{m}\right)\left(\varepsilon_{i}-\varepsilon_{m}\right)-\left(\varepsilon_{e}-\varepsilon_{m}\right)\left(\varepsilon_{i}+\varepsilon_{m}\right)\right|}{\left|Det\right|},$$

where the determinant Det is given by Equation (1). Det reaches a minimum when the ratio $\alpha =\alpha_{Res}$, given by Equation (2). The minimum of the determinant $Det=Det_{Res}$ (Equation (3)) corresponds to the maximum amplitude of the internal field, which is estimated as

(6) $$\left|A_{max}\right|=\frac{2a\varepsilon_{i}Q_{m}}{\left(\varepsilon_{e}+\varepsilon_{i}\right)}\left|\frac{{\varepsilon_{m}^{\prime }}^{2}-\varepsilon_{e}^{2}}{\varepsilon_{e}\varepsilon_{i}-{\varepsilon_{m}^{\prime }}^{2}}\right|∼Q_{m}$$

where it is assumed that the metal optical quality $Q_{m}\gg 1$.

**Figure 1:**
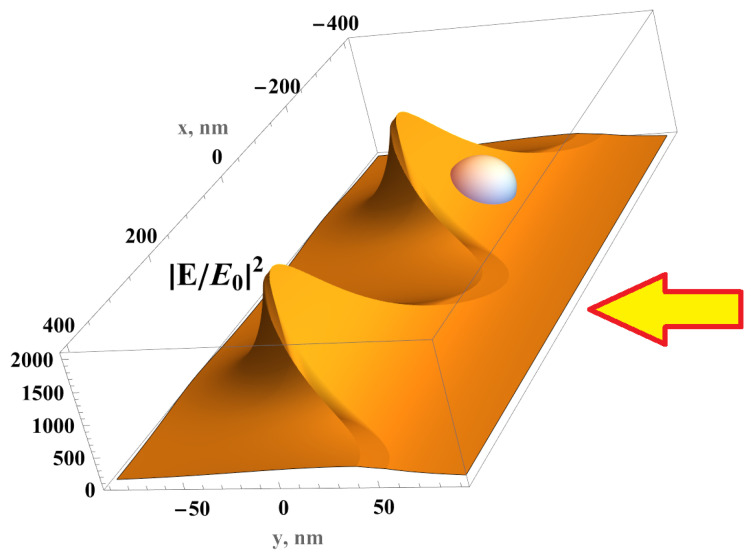
Electric field distribution in the silver FMS illuminated from the right (wavevector k$=\left\{0,-\omega /c,0\right\}$, $\varepsilon_{i}=1$) by light with amplitude $E_{0}$, which is polarized along the *x* direction; period of the FMS *L* = 400 nm, modulation *h* = 80 nm, silver thickness $d_{max}$ = 15 nm. Silver nanofilm is deposited on modulated polycarbonate; an exosome is schematically shown being immersed in a strong electric field in the depression of the FMS.

The conformal mapping (see [80,84,85,86,87])

(7) $$z_{1}=x_{1}+i\;y_{1}=-i\log\left(w-a\right),$$

transforms the metal NC into a modulated metal metasurface extending along the $x_{1}$ axis in the space $\left\{x_{1},y_{1}\right\}$. The dimensionless coordinates $\left\{x_{1},y_{1}\right\}$ are equal to $\left\{x_{1},y_{1}\right\}=\left(2\pi /L\right)\left\{x,y\right\}$, where *L* is the period of FMS modulation. The inner interface of the cylinder transforms into the right film surface $y_{u}\left(x\right)$, which is illuminated by the light falling from the right (from y=+∞), as shown in Figure 1.

The outer surface of the cylinder transforms into the left film surface $y_{b}\left(x\right)$. That is, the internal space of the cylinder (a<r<1) maps to the right half-space $y_{u}<y<+\infty$, and the external space (α<r<∞) maps to $-\infty <y<y_{b}$. The left $y_{u}\left(x\right)$ and right $y_{b}\left(x\right)$ surfaces of the FMS are given by the following equations:

(8) $$\begin{array}{ccc} y_{u} & = & -\frac{L}{2\pi }\log\left(\sqrt{1-a^{2}\sin^{2}x_{1}}-a\cos x_{1}\right), \end{array}$$

(9) $$\begin{array}{ccc} y_{b} & = & -\frac{L}{2\pi }\log\left(\sqrt{\alpha^{2}-a^{2}\sin^{2}x_{1}}-a\cos x_{1}\right), \end{array}$$

where $x_{1}=2\pi x/L$. The maxima of the right interface of the FMS have coordinates $x_{u,max}=\pm nL$, $y_{u,max}=-\left(L/2\pi \right)\log\left(1-a\right)>0$, and n=0,1,2… The depressions in the right interface of the FMS have coordinates $x_{u,min}=\pm nL+L/2$ and $y_{u,min}=-\left(L/2\pi \right)\log\left(1+a\right)<0$; recall the parameter 0<a<1. The same equations hold for maxima–minima of the left interface, and it is only necessary to replace 1 by α inside parentheses in log. We obtain that the modulation *h* of the FMS is equal to $h=y_{u,min}-y_{u,max}$ = (L/π)arctanha. The maximal thickness *d* of the FMS is equal to $d=y_{u}\left(x_{1}=0\right)-y_{b}\left(x_{1}=0\right)=\left(L/2\pi \right)\log\left[(\alpha -a)/(1-a)\right]$. Therefore, the ratio of the NC radii α and the position of the source *a* in the frame *w* can be obtained through the period *L*, the modulation *h*, and the thickness $d_{max}$ of the real FMS.

The external complex potential $U_{ext}\left(w\right)$ given by Equation (4) transforms into $U_{ext}\left(z\right)=-U_{0}\left(2\pi /L\right)z$ after the conformal mapping (7); that is, the FMS given by Equations (8) and (9) is excited by a constant electric field $E_{0}=-dU/dz=U_{0}\left(2\pi /L\right)$, which is directed along the *x* axis, i.e., along the FMS. The field $E_{0}$ corresponds to the excitation of the FMS by a plane wave normally incident on the FMS when the modulation *h* is much smaller than the wavelength λ. The amplitude of the electric field in front of the FMS, which is constant inside the NC in the *w* frame (see Equations (5) and (6)), transforms into the amplitude

(10) $${\left|E_{i}/E_{0}\right|}^{2}={\left|dw/dz_{1}\right|}^{2}{\left|A\right|}^{2}={\left|A\right|}^{2}\exp\left(-4\pi y/L\right)∼Q_{m}\exp\left(-4\pi y/L\right),$$

where the amplitude *A* is given by Equation (5). Therefore, we obtain a simple analytical solution for the amplitude of the local electric field in front of the FMS, which is illuminated by the light polarized normal to the FMS modulation. The field concentrates in the depressions, where the coordinate *y* is minimal (Figure 1). The ratio of the field in a depression and at the tops of the FMSs is equal to exp(−4πh/L), where *h* is the film modulation. The enhanced local electric field fills the depressions in the FMS, where exosomes are placed as shown in Figure 1.

**Figure 2:**
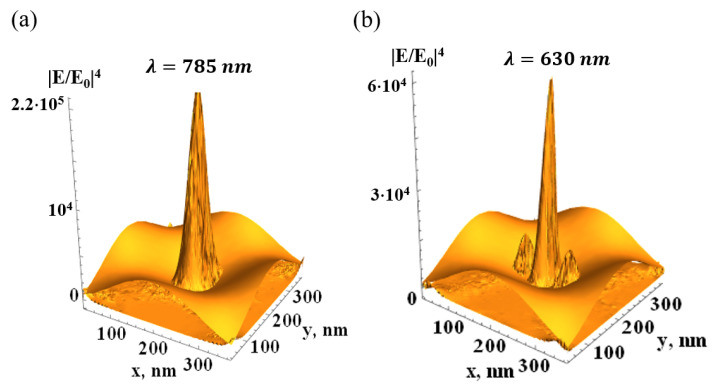
Distribution of the SERS factor $G\left(r\right)={\left|E\left(r\right)/E_{0}\right|}^{4}$ in the silver FMS illuminated from the top (wavevector $k=\left\{0,0,k\right\}$, $\varepsilon_{i}=1$) by light with amplitude $E_{0}$; periods of the FMS (shown thematically) *L* = 400 nm, other parameters as in Figure 1; (**a**)—dipole plasmon resonance, (**b**)—quadrupole resonance.

The local electric field is much enhanced upon plasmon resonance. The resonance field is estimated from Equations (6) and (10) as $E_{max}∼E_{0}Q_{m}$. The enhancement *G* of the Raman signal is proportional to $G∼\langle {\left|E_{max}/E_{0}\right|}^{4}\rangle ∼Q_{m}^{4}$. This is the SERS factor averaged over the MS (cf. derivation in [78]). However, the local electric field is nonuniform, and the RS of a molecule depends on its location on the surface. To gain insight into the SERS distribution, the local enhancement factor could be estimated as $G\left(r\right)∼{\left|E\left(r\right)/E_{0}\right|}^{4}$. The spatial distribution G(r) in the FMS presented in Section 3.3 is shown in Figure 2. The results were obtained from a full-scale COMSOL 4-1 computer simulation. In a real SERS experiment, the wavelength of the incident light is fixed according to the laser used and other factors of the experimental setup. Plasmonic resonance for a given period and modulation of the FMS is achieved by adjusting the thickness of the metal film deposited on top of the modulated dielectric substrate (see Section 2.2).

In this work, we used a 785 nm laser and silver FMS. The silver permittivity $\varepsilon_{Ag}\left(\lambda =785\;\mathrm{nm}\right)\approx -29.8+i0.38$ [83] has optical quality $Q_{Ag}=\left|ℜ\left(\varepsilon_{Ag}\right)/ℑ\left(\varepsilon_{Ag}\right)\right|\approx 80$; therefore, the SERS effect, obtained in the quasistatic approximation, can be as large as $G∼10^{7}$. The radiation loss decreases *G* in the real FMS, as shown in Figure 2. Quadrupole and higher resonances, arising from the expansion (4) of the external potential $U_{0}$ in the power series, can be considered in the same manner. However, the dipole resonance in the FMS seems to be the most suitable for the SERS experiment.

### 2.2. Flexible Metasurface Preparation

**Figure 3:**
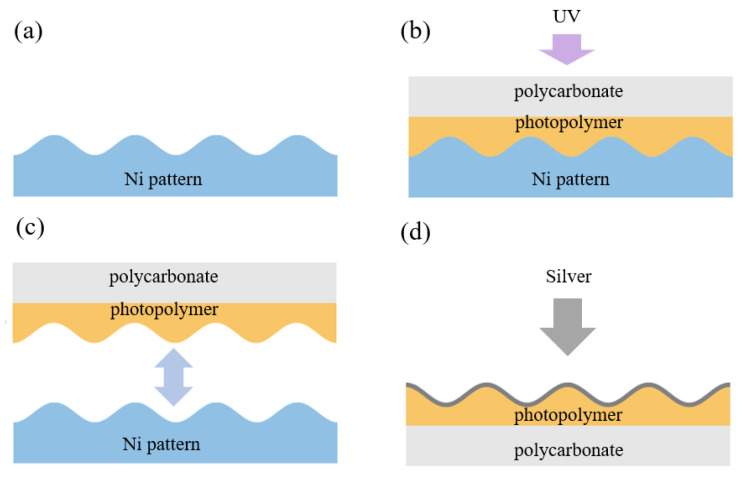
Process of manufacturing FMS via imprinting: a nickel matrix, obtained with photolithography, is used as a punch to form the photopolymer, which is then deposited on a thin polycarbonate substrate. FMS fabrication steps: (**a**,**b**) production of the modulated nickel pattern and the formation of a flexible polycarbonate substrate based on this pattern. A thin layer of photopolymer is applied to the modulated nickel, and a polycarbonate plate is placed on top. The assembly is then exposed to ultraviolet light for initial polymerization. (**c**) Separation of the nickel pattern from the photopolymer and polycarbonate substrate. (**d**) Deposition of the silver surface layer using electron-beam evaporation in a vacuum.

Flexible metasurfaces (FMSs) are produced using a holographic lithography technique in accordance with the technology described in our previous works [22,80], where a periodic structure was formed on a photopolymer film. The metasurface presented in the current study is a new modification fabricated via holographic interference lithography followed by silver deposition. The metasurface exhibits improved uniformity and overall surface quality, as confirmed by the AFM characterization shown in Section 3.3. In addition, the silver thickness in the current design was reduced to ∼20 nm since both the resonant optical response and the electric-field enhancement depend on the metal thickness. The manufacturing process for a double-period FMS has three steps: the production of the modulated nickel pattern with a sub-wavelength period, the creation of a flexible polycarbonate substrate based on this pattern, and the deposition of a silver nanolayer on the top of the modulated substrate. A chromium underlayer and liquid photoresist were applied to a glass wafer and then dried. The recording setup included a spatial light modulator HED-6001 (monochrome LCoS reflective display) and an ultraviolet laser (λ = 405 nm). Next, the wafer was developed in a solution of Microposit 303A developer and 15% DI water and dried using a centrifuge at 4000 rpm. The developed wafer was then placed in a galvanic bath to obtain a Ni pattern through electrolysis. Once the electrolysis was complete, the nickel layer was detached from the glass, and the remaining photoresist was washed off the nickel. A thin layer of photopolymer was applied to the modulated nickel, and a polycarbonate plate was placed on top; they were pressed together to ensure the photopolymer penetrated into the modulated nickel. The assembly was then exposed to ultraviolet light for initial polymerization. After the ultraviolet polymerization, the nickel pattern was separated, and the polycarbonate substrate with the structured photopolymer layer was exposed again to ultraviolet light for further hardening. The scheme of the imprinting process is presented in Figure 3.

The final step is the deposition of a silver surface layer using electron-beam evaporation in a vacuum. The thickness of the silver layer sputtered onto the transparent plate was estimated based on the optical transmittance of the test sample at a wavelength of 600 nm. During deposition, the transmittance decreased to almost 1%, corresponding to a silver thickness of 50 nm. On the structured polycarbonate surface, however, the silver layer was on average twice as thin and distributed unevenly. We fabricated the double-modulated FMS with a period *L* of 400 nm and a modulation amplitude of 80 nm, as shown in Section 3.3.

### 2.3. Exosome Preparation and Deposition onto Flexible Metasurfaces

A purified and desalted solution of extracellular exosomes was obtained from the cell line HEK293T (Human Embryonic Kidney 293T) in accordance with the protocol developed by Thermo Fisher Scientific Inc., Waltham, MA, USA 02451 (accessed on 1 January 2024). [https://assets.thermofisher.com/TFS-Assets/LPD/Application-Notes/protocol-cell-culture-exosome-whitepaper.pdf] and was kindly provided to us by the Laboratory of Genetically Encoded Molecular Tools, Shemyakin–Ovchinnikov Institute of Bioorganic Chemistry. An exosome stock solution (30 μL) was drop-coated on the FMS surface and covered with a cover glass for 10 s. The glass was then removed by slowly moving it along the FMS to its edge. The sample was then dried in a desiccator for 3 h.

### 2.4. Combined AFM/Microspectroscopy Measurement

Exosomes deposited on the surface of the FMS were examined with a custom-made setup. The system for probe-optical 3D correlative microscopy combines 2D confocal optical microspectroscopy and 3D scanning-probe microscopy in a single apparatus [88]. The 3D optical probe correlation microscopy system was developed in collaboration with scientists from the V.I. Shumakov Federal Scientific Center for Theoretical and Applied Physics of the Russian Ministry of Health, SNOTRA LLC, and the National Research Nuclear University MEPhI. The setup includes specially designed and optimized units: a sample surface modification system (ultramicrotome), a scanning-probe microscopy system, an optical unit with a confocal module, a Shamrock 750 monochromator (Andor) with a DU971P-BV CCD camera (Andor Technology, Belfast, Northern Ireland, GB), a tunable Ar laser, a Melles Griot 25-LHP-928-230 He-Ne laser, and a photodiode lasers (532,785 nm). All details can be found at [https://www.ibch.ru/about/unscieq] (in Russian, accessed on 31 January 2023). The SERS spectra were obtained using an upright confocal optical part of this setup that contains two Ultrasteep long-pass edge filters (RazorEdge and Semrock) to suppress exciting laser radiation and an objective lens (50× Mitutoyo Plan Apo HR Infinity Corrected Objective NA 0.75, WD 5.2 mm, Mitutoyo Corp, Kanagawa 213-8533, Japan). A 785 nm laser with a power of 1.5 mW was used. The measurements were carried out with an exposure time of 1 s and single accumulation. The SERS spectra were taken in the range of the Stokes shift $\Delta \omega_{s}=\omega -\omega_{s}$ from 600 to 1700 cm^−1^. The images obtained via atomic force microscopy (AFM) and scanning spreading resistance microscopy (SSRM) were acquired using the scanning-probe microscopy unit of the system for probe-optical 3D correlation microscopy using 1024 × 1024 pixels per scan with NSG10 and CSG10/Au (NT-MDT) probes, respectively. We used the semicontact mode in the case of AFM and the contact mode in the case of the combined AFM/SSRM. The AFM and optical microspectroscopy fields of view were aligned using laser confocal imaging of the probe tip. All SERS spectra were acquired after AFM mapping of a single exosome. We assume that our confocal alignment procedure yields a spectrum of an individual exosome that matches its AFM image.

### 2.5. Water-Immersed SERS Measurements

**Figure A2:**
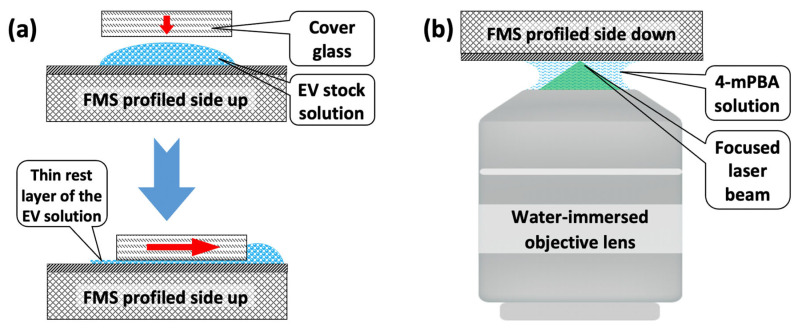
Explanations for the Materials and Methods section. (**a**) Schematic of exosome deposition on the FMS surface. Red arrows indicate the direction of motion of the cover glass. (**b**) Schematic of the water-immersed SERS measurements.

The original SERS measurement was performed in water to estimate the sensitivity of the FMS in terms of the limit of the detection (LOD) for a molecular monolayer covering the FMS surface. 4-Mercaptophenylboronic acid (4-mPBA, Sigma-Aldrich, St. Louis, MO, USA) was chosen as a model analyte because its small molecules form a covalently bonded monolayer with a thickness of around 0.8 nm on silver surface via thiol group bonding [89]. Covalent interaction of 4-mPBA with silver FMS increases the homogeneity of the surface distribution. The 4-mPBA was diluted with MQ-grade water to the concentrations 50, 5, 0.5, and 0.2 μM. An NtegraSpectra, Sutton, The Netherlands inverted Raman microspectrometer (NT-MDT) was used for the SERS experiment. The 4-mPBA solutions were drop-coated (30 μL) on the glass surface of the water-immersed Leica X63/NA = 0.9 objective lens, Leica Microsystems, Wetzlar, Germany (see Figure A2). The solution served as an immersion medium, whereas the objective lens was focused directly on the FMS. The 785 nm laser with a power of about 1.5 mW was used. The measurements were performed with an exposure time of 10 s and single accumulation. The SERS spectra were obtained in the Stokes range from 600 to 1700 cm^−1^. This approach suggests that the Raman enhancement value depends only on the properties of the metasurface itself rather than on fluctuations in the surface concentration of the analyte.

## 3. Results

### 3.1. Mathematical Model of Optical FMS

The interaction of the incident laser beam with the FMS was investigated by means of the developed mathematical model. Our simple mathematical model of a unidirectional modulated FMS was used to calculate the local optical electric field in an FMS illuminated by an electromagnetic wave in an SERS experiment. Explicit equations give the local field. The spatial distribution of the field is counterintuitive since the electric field is concentrated in the depressions of the modulated FMS, as shown in Figure 1. The maximum electric field is found as a function of the period *L*, modulation *h*, and thickness *d* of the metal nanofilm. The film was deposited on a flexible plastic substrate. We assessed how to achieve the possible maximum of the local field $E_{max}$ for a given period and modulation amplitude. The electric field at the bottom of the film depression was obtained based on Equations (8) and (10).

(11) $$\left|\frac{E_{max}}{E_{0}}\right|≃\frac{2\varepsilon_{i}Q_{m}a\left(a+1\right)}{\varepsilon_{e}+\varepsilon_{i}}\exp\left(-\frac{2\pi \Delta y}{L}\right),\;\;\;a=\tanh\left(\frac{\pi h}{L}\right),$$

where $E_{0}$ is the electric field of the incident light, Δy≥0 is the distance from the bottom of the FMS depression, $\varepsilon_{i}$ is the permittivity of the external medium (r.h.s. in Figure 1, upper half space in Section 3.3, and lower half-space in Figure A2), $\varepsilon_{e}\approx 2.5$ is the permittivity of the polycarbonate substrate of the FMS, and metal quality $Q_{m}=|\varepsilon_{m}^{\prime }|/\varepsilon_{m}^{\prime \prime }\gg 1$. We assume that the film thickness *d* has an optimal value $d_{max}$ that is estimated as $d_{max}≃\left(L/2\pi \right)\left(\varepsilon_{e}+\varepsilon_{i}\right)/\left|\left(1-a\right)\varepsilon_{m}^{\prime }\right|$ in the case $\left|\varepsilon_{m}^{\prime }\right|\gg 1$. In the bottom of the FMS depressions, the metal is thinner, and it is estimated as $∼\exp\left(-2\pi h/L\right)d_{max}$. Recall that $\varepsilon_{Ag}\left(\lambda =785\;\mathrm{nm}\right)≃-30+i0.38$ [83]. The permittivity of the front half-space $\varepsilon_{i}$ is equal to one when the upper surface of the FMS is “free”, and the permittivity $\varepsilon_{i}$ is equal to the optical permittivity of water for the water-immersed SERS measurement discussed in Section 2.5. Substituting the silver parameters into Equation (11), we find that for the silver FMS, the local electric field could be two orders of magnitude larger than the field of the incident electromagnetic wave.

A double-period silver FMS was used in our experiment as the SERS substrate (see Figures in Section 3.3). The mathematical model allows a qualitative understanding of the origin of the plasmon resonance, and the model estimates the optimal parameters. A comprehensive discussion of the computer simulation of the double periodic FMS is presented in our previous paper [22]. The computer results correspond well to the mathematical model. The significantly enhanced local electric field is indeed concentrated in the depressions of the FMS; it fills the volume of ∼(*L*/2 ${\pi )}^{3}$, where exosomes are placed for excitation and obtaining the Raman signal.

### 3.2. Estimation of FMS Sensitivity for Liquid Analyte at Adsorption/Desorption Equilibrium

**Figure 4:**
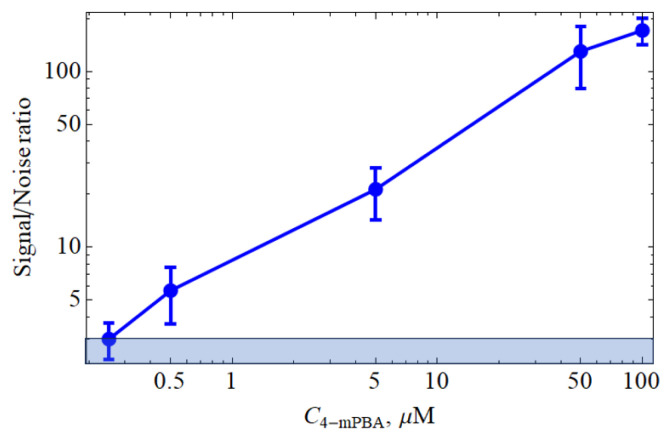
SERS intensity versus 4-mPBA concentration, where the blue zone corresponds to the zone of the 3× noise level.

We determined the signal/noise dependence for the SERS spectra obtained for aqueous solutions of 4-mPBA with concentrations of 50, 5, 0.5, and 0.05 μM (see Figure 4). As described in Section 2.5, the examined solutions served as an immersion medium. The objective lens was focused directly on the FMS surface. The intensity of the band in the region of 1589 cm^−1^ served as the signal parameter, and the average amplitude of the baseline noise was used as the noise parameter. It was found that the signal/noise value for the SERS spectra depends linearly on the concentration of 4-mPBA and at its value of 0.2 μM reaches a value of 3, which can be considered as the LOD for our amplifying systems with the experimental parameters described in Section 2.5.

### 3.3. Exosomes Deposited on FMS

**Figure 5:**
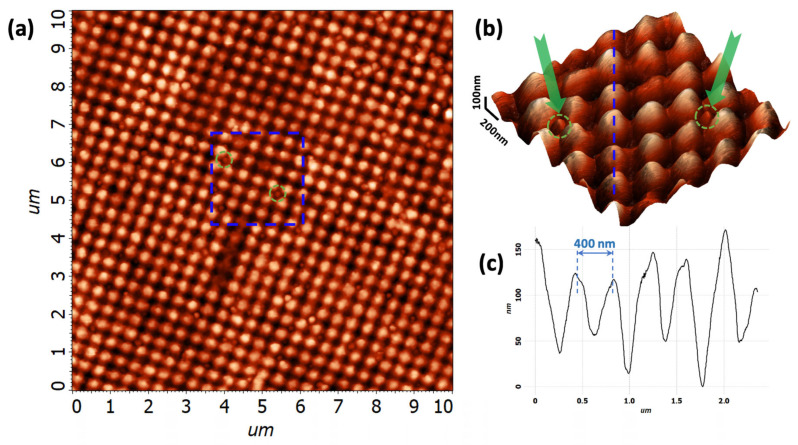
Atomic-force microscope image of the exosomes deposited onto the FMS surface. (**a**) AFM image of a 10×10μ FMS area with a highly rarefied monolayer of exosomes on the FMS surface. The blue dashed square marks the zoomed area containing two exosomes marked by the green dashed circles; (**b**) 3D-AFM image of a 2×2μ area marked in panel (**a**). The blue dashed line marks the band along which the cross-section was created, and the arrows and dashed circles mark individual exosomes; (**c**) Cross-section along the blue dashed line on panel (**b**) showing modulation of the FMS.

Figure 5 shows an AFM image of a 10×10μ FMS area with a highly rarefied monolayer of the exosomes on the FMS surface. Exosomes deposited on the metasurface are easily identified as point disturbances of the ordered structure of the metasurface. As an example, two of them are marked in Figure 5a with green circles. Figure 5b shows a 3D-AFM image of a 2×2μ region marked in Figure 5a. This image clearly shows that single exosomes (marked with the green arrows and dashed circle) are well suited for the deposition in the center of a metasurface depression. In addition, this image allows us to estimate the parameters of the FMSs themselves. Thus, in Figure 5b, a sequence of periodic FMS structures (marked with a blue dotted line) along which both exosomes and other imperfections are absent was selected. A cross-section was created along this sequence, as shown in Figure 5c. The data in this figure allow us to estimate the amplitude of the modulation of the resulting structures. The FMS height swings between the maximum and minimum height in the range from 60 to 170 nm. The average amplitude of the modulation *h* is in the range of h=70÷90 nm. Note that the period of the modulation *L* is much more uniform and is equal to L=400±10 nm.

**Figure 6:**
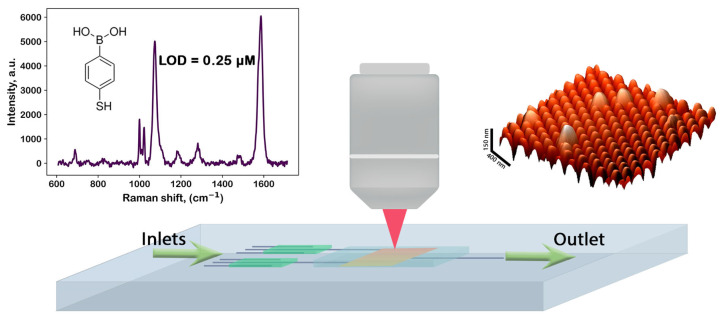
Concept of a Lab-On-Chip (LOC) analytical system based on flexible metasurface (FMS); AFM image of FMS is shown in upper left panel in brown color, white spheres schematically show some microobjects, deposited on FMS.

Our researched and developed FMS can be manufactured in a wide range of sizes and shapes, which allows it to be easily integrated into a Lab-On-Chip system as a spectral detection chamber (see Figure 6). The prototype of such an LOC system is discussed in Section 3.2 (see also Appendix A). The LOD of such systems was determined (Figure 4 and Figure 6, top-left panel) to be 0.2 μM for molecules under standard experimental conditions described in Section 2.5. Note that the FMS does not distort the original Raman signal in the process of SERS. Moreover, the FMS has a uniform gain over the entire scanning area, in contrast to most other random substrates where the SERS signal is obtained from so-called hotspots (see, e.g., [77,89]). These distinguishing properties of the FMS allow one to consider the FMS as a basis for the SERS detection chamber in modern microfluidic systems. It is also worth noting that the FMS is an excellent surface spatial filter for exosomes and other vesicle-like objects (see Figure 5, top-right panel).

### 3.4. Single-Exosome SERS

The combined AFM/microspectroscopy technique is used to identify single exosomes and record their SERS spectra. The main purpose of this combined approach is to obtain the SERS spectra from single exosomes. Confocal scanning can also be used for this purpose, but this method cannot differentiate between single objects and agglomerates of objects similar in size to an exosome. At the same time, the use of AFM allows us to determine single exosomes with high accuracy based on their morphology. Taking into account that the AFM and optical microscopy fields of view are aligned according to the technique described in Section 2.4, we can further state that the recorded SERS spectra correspond to single exosomes determined via AFM. However, there is a difficulty in that the FMS surface has a highly developed structure, and at the same time, there are imperfections on it that are topographically similar to exosomes. In the vast majority of cases, such imperfections arise during the fabrication of the periodic polymer matrix before the silver deposition. The localized imperfections arising after silver deposition can be caused by external contamination. This contamination can be minimized by combining all FMS production processes in one clean room.

**Figure 7:**
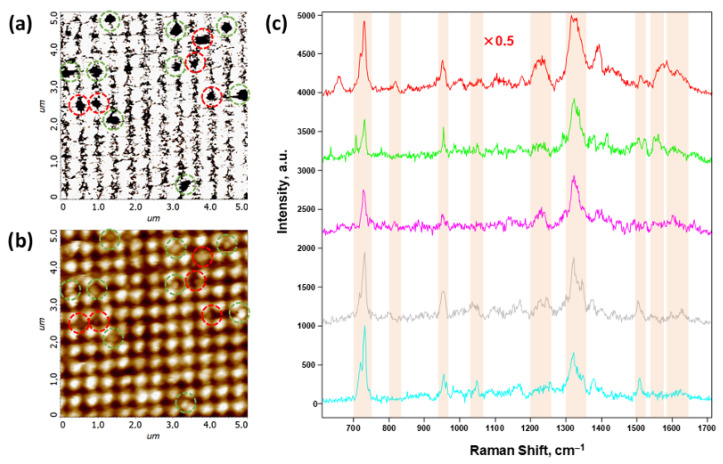
Correlated scanning spreading resistance microscopy (SSRM), atomic force microscopy (AFM), and surface-enhanced Raman scattering (SERS) data of exosomes deposited onto the FMS surface. (**a**) SSRM image of a 5 × 5 μm section of the FMS with a highly rarefied monolayer of the exosomes on the FMS surface; (**b**) AFM image of the same section as in panel (**a**); (**c**) SERS spectra of the exosomes obtained at the points marked with the green dashed circles on panels (**a**,**b**). Green and red dashed circles on panels (**a**,**b**) mark the putative exosomes and imperfections, respectively; (**c**) SERS spectra of five exosomes are shown by different colors, the upper spectrum is normalized by a factor of 0.5; spectral bands marked on panel (**c**) are assigned in Table 1.

Thus, it can be concluded that the main difference between exosomes and morphologically similar imperfections is that exosomes do not conduct current. This difference is rather important from the point of view of the scanning-probe microscopy methods developed in this work. This circumstance makes it possible to identify exosomes much more accurately using the correlative combination of SSRM and AFM. Figure 7 shows an example of such a methodical combination, where Figure 7a,b show the SSRM and AFM data, respectively, of the same FMS section with exosomes deposited onto its surface. The most significant areas, where the conductivity current value drops significantly, are marked with dashed circles in the panel (Figure 7a). The grid of these circles was transferred to the complementary AFM image in Figure 7b. It was revealed that in some areas marked in this way, there are no significant topographic features. These “zero”-conductivity points are most likely due to defects in the silver deposition. Such areas are marked with red circles in both panels. Figure 7c shows the most typical SERS spectra obtained from the points marked with green circles in Figure 7a,b, which we attributed to a single exosome. The obtained spectra are in a correlation with those obtained earlier in [90,91,92], and the results of their analysis taking into account the literature data (see, e.g., [1]) are presented in Table 1. Typical SERS spectra from “waste” points marked by red circles are shown in the figure in Appendix A.

## 4. Discussion

We investigated a flexible metasurface (FMS) that accumulates and enhances the electric field of incident light. FMS is a doubly modulated silver film with a period *L*, which is approximately half the wavelength. The field is enhanced by collective plasmon resonances excited in the FMS. A distinctive feature of the FMS is the spatial scale ξ∼L of the enhanced electric field. The size η of the exosome under study is smaller than η<ξ. Consequently, all exosome molecules interact with the enhanced electromagnetic field and cause the RS. Let us assume that the local electric field is enhanced tenfold only. Then, the Raman signal from one exosome deposited at the FMS is equivalent to the Raman signal from $10^{4}$ free exosomes.

The study of the researched and developed FMS via AFM shows that the production method accurately reproduces the period of the modulation L=400±10 nm. However, a significant spread in the amplitude of modulation is observed in Figure 5c. We speculate that the heterogeneity in the FMS modulation is not a significant drawback of such a structure when being employed as an exosome sensor. Indeed, exosomes usually have a size of η∼30÷150 nm. Exosomes are formed via the endosomal pathway and are mainly of interest for this area of research as biomarkers. Therefore, they are morphologically complementary to the FMS in the entire range of their sizes. Even in the case of modulation heterogeneity, the FMS effectively adsorbs exosomes in the form of a single exosome per depression, as shown in Figure 5b. The proposed approach combines the SSRM, AFM, and SERS techniques and allows us to significantly increase the reliability of the identification of single exosomes. The regular FMS profile is best suited for identifying individual exosomes compared to most other disordered SERS substrates. The SSRM method allows us to cut off the overwhelming majority of micro inhomogeneities above the periodic structure of the FMS at the first step. Then, the task that remains for AFM analysis is to determine whether the shape of the remaining candidates resembles that of an exosome and to determine whether the identified object is a single exosome or a cluster of them.

## 5. Conclusions

The FMS presented in the work has significant potential as a basis for an analytical system based on recording the SERS spectra of exosomes and other vesicle-like objects since it has a uniform gain over the scanning area, does not distort the analyte Raman spectrum, and has high morphological complementarity to such objects. These distinguishing properties open up prospects for using such an FMS as a basis for creating an SERS registration module in automated LOC systems. Moreover, an FMS-based LOC system can be proposed for the detection and recognition of micro- and nanoplastics, viruses, spores, and any other micro or nano objects. In addition, the proposed synthetic approach, combining SSRM, AFM, and SERS techniques, can significantly improve the efficiency of research methods for identification and spectral study of individual exosomes using SERS spectroscopy. 

## Figures and Tables

**Table 1:** Assignment of the major bands in the exosome SERS spectra marked in Figure 7.

Raman Shift, cm^−1^	Assignment
715–740	Tryptophan, coenzyme A, nucleic acids
810–830	Tyrosine
940–965	α-Helix backbone ν(C-Cα-N)
1025–1060	C-N, C-C stretching of protein and lipids
1200–1265	Amide III proteins, nucleic acids U
1300–1370	$CH_{2}-CH_{2}$ of nucleic acids, Protein Backbone δ(CαH)
1495–1530	Carotenoids polyene ν(CvC)
1545–1570	Nucleic acids (guanine), amide II, Trp proteins
1575–1630	Trp, Tyr, and Phe Proteins

**Figure A1:**
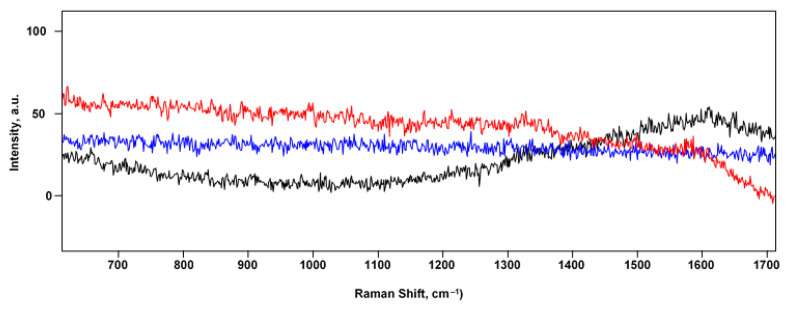
SERS spectra, shown in red, blue, and black colors, are obtained by focusing the confocal Raman spectrometer on three FMS points marked with red circles in Figure 7.

## Data Availability

The original contributions presented in this study are included in the article. Further inquiries can be directed to the corresponding author.

## Abbreviations

The following abbreviations are used in this manuscript:

4-mPBA	4-Mercaptophenylboronic Acid
AFM	Atomic Force Microscopy
EV	Extracellular Vesicle
FMS	Flexible Metasurface
LOC	Lab-On-Chip
LOD	Limit of Detection
MS	Metasurface
NC	Nanocylinder
NP	Nanoparticle
RS	Raman Scattering
SERS	Surface-Enhanced Raman Spectroscopy
SSRM	Scanning Spreading Resistance Microscopy
